# Comparative transcriptomic analysis of the different developmental stages of ovary in the cuttlefish *Sepia pharaonis*

**DOI:** 10.1186/s12864-024-09981-x

**Published:** 2024-01-23

**Authors:** Maowang Jiang, Qingxi Han, Liting Xu, Ruibing Peng, Tao Zhang, Xiamin Jiang

**Affiliations:** 1 Key Laboratory of Applied Marine Biotechnology, School of Marine Sciences, Ningbo University, 818 Fenghua Road, Ningbo, 315832 Zhejiang Province PR China; 2 Marine Fisheries Research Institute of Zhejiang Province, Zhoushan, 316022 China

**Keywords:** Transcriptomics, Ovary, Molecular mechanisms, Reproductive regulation, *Sepia pharaonis*

## Abstract

The cuttlefish, *Sepia pharaonis*, is characterized by rapid growth and strong disease resistance, making it an important commercially farmed cephalopod species in the southeastern coastal regions of China. However, in the reproductive process of *S. pharaonis*, there are challenges such as a low output of eggs, poor quality, and low survival rates of newly hatched juveniles. Therefore, there is an urgent need to study the molecular mechanisms underlying ovarian development in this species. In this study, we conducted the first transcriptomic analysis of the ovary at four developmental stages: the undeveloped stage, developing stage, nearly-ripe stage, and ripe stage, and compared the transcriptomics among these four stages using Illumina sequencing technology. The total numbers of clean reads of the four stages ranged from 40,890,772 to 52,055,714 reads. A total of 136,829 DEGs were obtained, GC base ratios of raw data were between 38.44 and 44.59%, and the number of uniquely mapped reads spanned from 88.08 to 95.90%. The Pearson correlation coefficient demonstrated a strong correlation among different samples within the same group, PCA and Anosim analysis also revealed that the grouping of these four stages was feasible, and each stage could be distinguished from the others. GO enrichment analysis demonstrated that ovarian follicle growth, sex differentiation, and transforming growth factor beta receptor, played a foreshadowing role at the early ovarian development stage, and the terms of small molecule metabolic process, peptide metabolic process, and catalytic activity were prominent at the mature stage. Meanwhile, KEGG analysis showed that the early ovarian development of *S. pharaonis* was mainly associated with the cell cycle, DNA replication, and carbon metabolism, while the mid-late ovarian development was involved with the signal transduction, endocrine system, and reproduction pathway. RT-qPCR further confirmed the consistent expression patterns of genes such as *17β-HSD*, *GH*, *VGS*, *NFR*, and *NYR* in the ovaries of *S. pharaonis*, exhibiting elevated levels of expression during the maturation stage. Conversely, *ER* and *OM* exhibited high expression levels during the early stages of ovarian development. These transcriptomic data provide insights into the molecular mechanisms of *S. pharaonis* ovarian development. The findings of this study will contribute to improving the reproduction and development of cuttlefish and enriching the bioinformatics knowledge of cephalopods.

**Supplementary Information:**

The online version contains supplementary material available at 10.1186/s12864-024-09981-x.

## Introduction

The cuttlefish *Sepia pharaonis* is a broadly distributed neritic demersal cephalopod species that inhabits shallow coastal areas at depths ranging from the nearshore to 130 m [1]. This species has an annual life cycle. Adult individuals migrate nearshore, congregate in offshore waters for mating, and lay their eggs in shallow waters (5–20 m) during a breeding period that spans from January to March [2]. In recent years, *S. pharaonis* has been successfully bred under captive conditions, achieving a growth period of four months in cement tanks and reaching weights exceeding 500 g. This species exhibits rapid growth and disease resistance characteristics [3]. Additionally, individuals have attained weights exceeding 2.2 kg in annual aquaculture cycles, establishing it as a commercially important cephalopod species in the southeastern coastal regions of China [4].

Due to its high economic value and potential for aquaculture, *S. pharaonis* has attracted considerable interest. Previous studies on *S. pharaonis* mainly focused on biology and optimization of culture conditions [5–7], to date, little information has been available on the nature of gene transcripts involved in *Sepia* gonad development and reproductive cycle [8]. Our recent studies have demonstrated that ovarian development in *S. pharaonis* is not synchronous and can be categorized into five stages: the non-developmental stage, the developmental stage, the near-maturity stage, the maturity stage, and the degeneration stage [9]. However, in the course of conducting a comprehensive study on the full-scale captive breeding of *S. pharaonis*, we identified several challenges that limit the scale of captive breeding for this species. These challenges include a low yield of eggs, poor egg quality (with a hatching rate below 30%, compared to over 85% in the wild) [4], as well as frail physical conditions in newly hatched juveniles leading to low survival rates. These challenges have become crucial bottlenecks in the full-scale captive breeding of the species [4]. Therefore, conducting transcriptome sequencing during gonad differentiation may help identify the reproductive mechanisms in *S. pharaonis*, contributing to the improvement of captive breeding technologies.

Transcriptomic analyses of cuttlefish ovaries provide contemporary insights into breeding fertility and contribute significantly to the field of developmental biology [10–12]. Molecular investigations into the ovaries of female *Sepiella* primarily focus on the transcriptomic analysis of both immature and mature developmental stages [8, 9]. Many genes, such as those encoding vitellogenin, vitellogenin receptor, GnRH receptor, estrogen receptor, and FMRFamide receptor, have been identified and demonstrated to have important roles in ovary maturation [10, 13, 14]. In addition, the advancement of molecular biology, sequencing and bioinformatics technologies offers a platform for the measurement of large-scale gene expression patterns and differential gene screening using high-throughput RNAsequencing (RNA-seq) [15]. Transcriptome sequencing serves as a low-cost and time-efficient approach for screening genes related to ovarian development, as well as other causative genes. Identifying the pathways implicated in gonad development can offer further insights into the gene regulatory networks governing ovarian development and, consequently, help maintain healthy ovarian function [16].

In this study, we utilized high-throughput sequencing to obtain the ovarian transcriptomes of female *S. pharaonis* across four stages of ovarian maturation. Differentially expressed genes (DEGs) between the stages were identified and subjected to enrichment analysis to elucidate the genes and pathways regulating ovarian maturation. These data provided new insights for understanding the mechanisms of reproductive regulation in cuttlefish. Furthermore, they provide a theoretical foundation for addressing challenges in *S. pharaonis* aquaculture, such as low egg production, poor egg quality, the weak constitution of hatchlings, and low survival rates.

## Materials and methods

### Animal culture and ovarian tissue collection

**Fig. 1:**
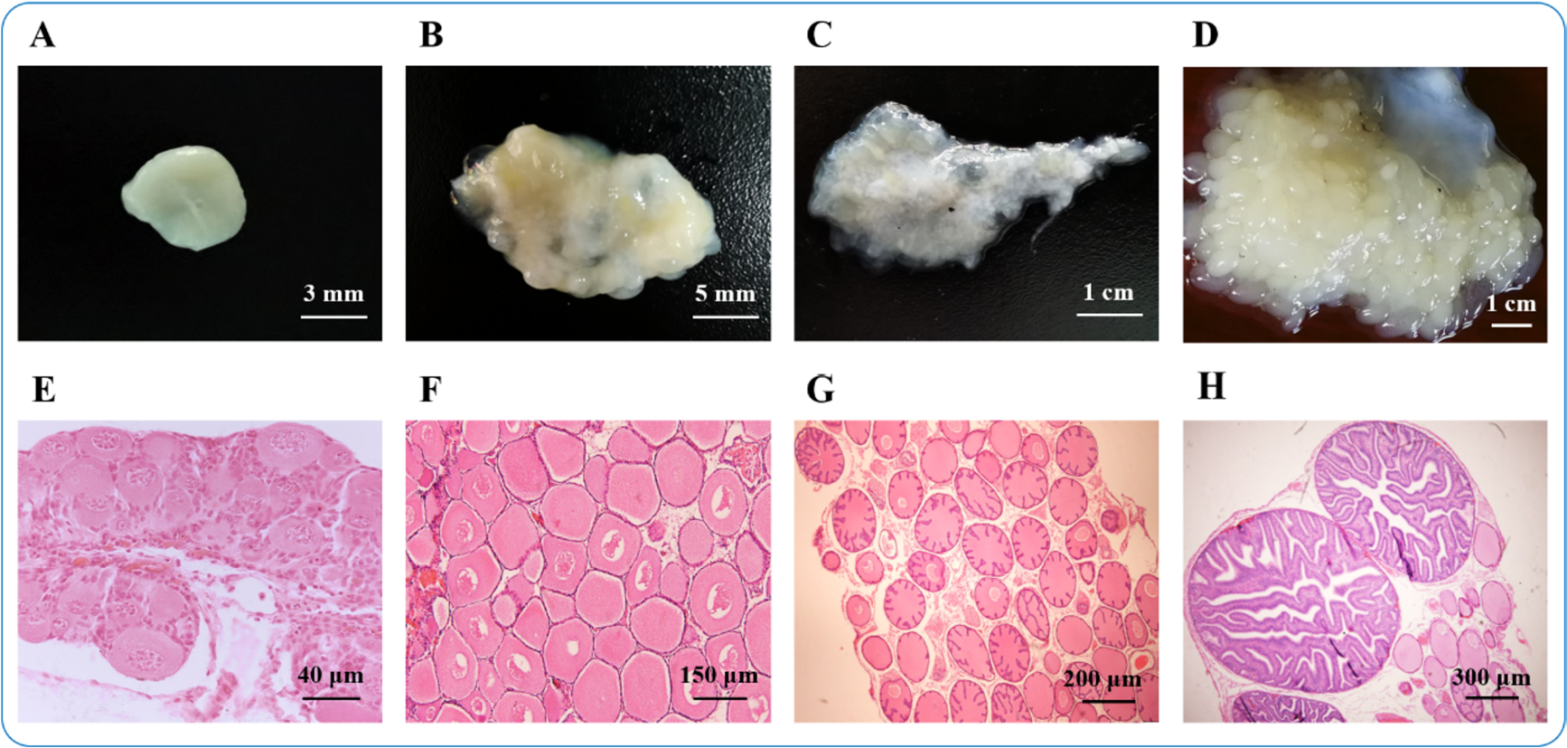
Identification of ovaries at different developmental stages of *S. pharaonis*. **A**-**D **The morphology of ovaries at different stages by photograph; **A **The morphology of the ovary at stage I, **B **The morphology the ovary at stage II, **C **The morphology of the ovary at stage III, **D **The morphology of the ovary at stage IV. **E**-**H **The histomorphology of ovaries at different stages by HE staining; **E **The oocytes at stage I, **F **The oocytes at stage II, **G **The oocytes at stage III, **H **The oocytes at stage IV

Adult cuttlefish (*S. pharaonis*) were reared at Lai Fa Aquaculture Co. Ltd in Zhejiang Province, China (29°59’ N, 121°99’ E), following the methodology outlined by Jiang et al. (2022) [17]. After mating, egg clusters were collected and incubated. For continuous sampling, the newly hatched animals are then cultured to sexual maturity after the fertilized eggs are laid, until they mate and lay eggs again. Experimental animals were fed in accordance with the methods described by Jiang et al. (2018) [3]. Ovarian staging was conducted based on the criteria established in our most recent study [8]. According to the incubation period, the ovaries were assigned to maturation classes by their morphology and color: Ova I (transparent, undeveloped stage), Ova II (milky white in strips, developing stage), Ova III (milky white eggs of different sizes, nearly-ripe stage), Ova IV (milky white like a bunch of grapes, ripe stage) (Fig. 1A-D). Eight specimens were randomly selected from each of the four ovarian maturation stages, and this was replicated four times for each stage. To corroborate the morphological classifications, a tissue section from each ovary was selected for hematoxylin and eosin (HE) staining (Fig. 1E-H). Ultimately, four samples from each stage were designated as follows: Ova I (I_1, I_2, I_3 and I_4), Ova II (II_1, II _2, II _3 and II_4), Ova III (III_1, III_2, III_3 and III_4), and Ova IV (IV_1, IV_2, IV_3 and IV_4). Ovarian tissues were immediately immersed in RNA preservation buffer (#R0118, Beyotime, China), flash-frozen in liquid nitrogen, and stored at -80 °C until subsequent analysis.

### RNA extraction, library construction and Illumina sequencing

Total RNA was extracted from the ovarian tissue using TRIzol® Reagent (Plant RNA Purification Reagent for plant tissue) according the manufacturer’s instructions (Invitrogen) and genomic DNA was removed using DNase I (TaKara). Subsequently, RNA quality was assessed using a 2100 Bioanalyzer (Agilent), and quantification was performed with an ND-2000 (NanoDrop Technologies). Only high-quality RNA samples (OD260/280 = 1.8 ~ 2.2, OD260/230 ≥ 2.0, RIN ≥ 6.5, 28 S:18 S ≥ 1.0, > 1 µg) were utilized for library construction. The RNA-seq transcriptome library was prepared using the TruSeq™ RNA Library Prep Kit for Illumina (San Diego, CA, USA) according to the manufacturer’s protocol. Initially, messenger RNA was isolated using the polyA selection method with oligo(dT) beads, and then fragmented using fragmentation buffer. Subsequently, double-stranded cDNA was synthesized using the SuperScript double-stranded cDNA synthesis kit (Invitrogen, CA) with random hexamer primers (Illumina). The synthesized cDNA was then subjected to end-repair, phosphorylation, and ‘A’ base addition following Illumina’s library construction protocol. Libraries were size-selected for cDNA target fragments of 300 bp on 2% Low Range Ultra Agarose, followed by PCR amplification using Phusion DNA polymerase (NEB) for 15 PCR cycles. After quantification with TBS380, the paired-end RNA-seq sequencing library was sequenced using the Illumina HiSeq xten sequencer (2 × 150 bp read length).

### Read mapping

The raw paired end reads were trimmed and quality controlled by SeqPrep (https://github.com/jstjohn/SeqPrep) and Sickle (https://github.com/najoshi/sickle) with default parameters. Subsequently, the clean reads were aligned to the reference genome using an orientation mode in HISAT2 (http://ccb.jhu.edu/software/hisat2/index.shtml) software [18]. For each sample, mapped reads were assembled using a reference-based approach in StringTie (https://ccb.jhu.edu/software/stringtie/index.shtml? t = example) [19].

### Differential expression analysis and functional enrichment

To identify differentially expressed genes (DEGs) between samples, expression levels for each transcript were calculated using the transcripts per million methods. RSEM (http://deweylab.biostat.wisc.edu/rsem/) was used to quantify gene abundances [20]. Essentially, differential expression analysis, functional annotation, and functional enrichment were performed according to the gene expression levels in different samples. Differential gene screening criteria were |log2 (fold change) |> 1 and padj < 0.05. GO and KEGG pathway enrichment analysis of the DEGs were performed, and the screening criterion was padj < 0.05 [21, 22]. In this study, the expression patterns of four developmental stages were revealed, and up-regulated/down-regulated genes were determined by comparing groups.

### RT‑qPCR verification of expression patterns

Eight candidate DEGs (Table 1) were selected to verify the reliability of RNA-seq data by RT-qPCR. We analyzed the expression profiles of these eight genes in four ovarian maturation stages. The primers for these genes (Table 2) were designed using Primer Premier (v 5.0) [23]. The PrimeScript™ RT reagent Kit with gDNA Eraser (TaKaRa) was used for cDNA synthesis following the manufacturer’s instructions. The qRT-PCR reaction was performed on a 7500 Real-Time PCR System (Applied Biosystems, Foster City, CA, USA), utilizing the SYBR Premix Ex Taq Kit (RR420A, TaKaRa, Dalian, China) in accordance with the manufacturer’s protocol. The fluorescent quantitative PCR reaction solution consisted of 10.0 µL SYBR® premix Ex TaqTM (2×), 0.5 µL PCR forward primer (10 µM), 0.5 µL PCR reverse primer (10 µM), 2.0 µL RT reaction mix (cDNA solution), and 7.0 µL dH_2_O. The reactions employed a two-step method, the qRT-PCR was performed in a volume of 20 µL under the following conditions: 95 °C for 10 min, followed by 40 cycles of 95 °C for 30 s, 95 °C for 5 s, and 60 °C for 20 s. Each gene was amplified in triplicate, generating dissociation curves for four stages of ovarian development. The specificity of the amplification was subsequently verified through melt curve analysis. The β-actin gene was used as an endogenous reference gene; each reaction is subjected to three biological replicates and three technical replicates, respectively. The expression levels of the target genes were calculated using the 2^–ΔΔCT^ method, as described by Livak and Schmittgen (2001) [24]. The β-actin gene served as the endogenous reference, and its expression level in the control group was set to 1.

**Table 1:** GO annotation and KEGG enrichment are involved in regulating reproduction and endocrinology

KEGG pathway	DEGs	Candidate genes	GO ID	KEGG ID
Steroid hormone biosynthesis	gene-SPHA_30631	Estradiol 17-beta-dehydrogenase	GO:0004303	map00140
	gene-SPHA_11793	Estrogen receptor	GO:0030331	map04915
	gene-SPHA_33362	Progesterone receptor	GO:0050847	map07226
GnRH signaling pathway	gene-SPHA_35598	Growth hormone	GO:0000187	map04935
	gene-SPHA_72978	Oocyte meiosis	GO:0030054	map04114
Ovarian steroidogenesis	gene-SPHA_30631	Estradiol 17-beta-dehydrogenase	GO:0004303	map00140
	gene-SPHA_27337	Vitellogenesis	GO:0007296	map04913
Neuroactive ligand-receptor interaction	gene-SPHA_20675	Neuropeptide F receptor	GO:0042263	K04209
	new_gene1033	Neuropeptide Y receptor	GO:0004983	K04204

**Table 2:** The sequences information of primers used for RT-qPCR

DEGs	Gene name	Primer sequences (5’ to 3’)	Product size (bp)	Amplification efficiency (%)
Estradiol 17-beta-dehydrogenase	*17β-HSD*	F: TCCTCTTTCTCACACGCTTTCTCTC	85	99.2
		R: AAGTCTGCCAAGTTCCTCATCTACC		
Estrogen receptor	*ER*	F: TGCCTCCTACCAATACTGCTGAAG	110	95.4
		R: GCTATGCTACGCCTGCTCACC		
Progesterone receptor	*PR*	F: ACTGTGTTTGACTGGGCAAGAAATG	126	97.7
		R: CGTGGAGTTGAGCATTGGACATTC		
Growth hormone	*GH*	F: AAATCCGTGTTGGTGATGGTCAAAC	126	101.8
		R: AGTCAGTTCCACAGTGTAGCAGTTC		
Oocyte meiosis	*OM*	F: GGTGAGACGCCCATCCTTTCC	141	98.5
		R: TCGTTTCCTCAATCCTCCCTGTTC		
Vitellogenesis	*VGS*	F: TGCCGTCACCTCCCTCTGTC	118	97.3
		R: GCGAGATGCCAGCCTAGTAGAAC		
Neuropeptide F receptor	*NFR*	F: CGCTGCTGTTGTTGGTGTATCTG	83	101.4
		R: CCTTGGACACTAGGTAGCCGATTC		
Neuropeptide Y receptor	*NYR*	F: TTAACCTGCCTCTGTGCGATGG	120	102.6
		R: GAGAAACGGCGAACTGCTTGTG		
	β-actin	F: GACTCCTACGTAGGAGACGA	235	98.6
		R: CGTTGAAGGTCTCGAACATGA		

### Statistical analysis

The RT-qPCR experiment was performed triplicate, and the data were presented as mean ± S.D. The statistical analysis was carried out based on one-way ANOVA. Gene expression histograms were drawn in GraphPad Prism 8.0.2 (https://www.graphpad.com/scientific-software/prism/). Multi-dimensional Venn diagrams were created using UpSetR plot to determine the overlapping results of comparison groups [25].

## Results

### Overview of the ovarian transcriptome of *S. pharaonis* at different ovarian development stages

Sixteen ovary samples from *S. pharaonis* in four different developmental stages (stage I: Ova I_1 to Ova I_4, stage II: Ova II_1 to Ova II_4, stage III: Ova III_1 to Ova III_4, and stage IV: Ova IV_1 to Ova IV_4) were subjected to RNAseq. In each stage, four biological replicates were performed to eliminate intra-group errors. The total raw read counts of all 16 samples ranged from 41,091,122 (Ova IV_4) to 52,488,460 (Ova II_2) (Table 3). Following quality control, adapters and low-quality reads were removed from the dataset. The cleaned data ranged from 40,890,772 (Ova IV_4) to 52,055,714 (Ova II_2) reads (Table 3). The GC base ratios of raw data were between 38.44 and 44.59%, with an average of was 40.29%. The sequence reads were mapped to the cuttlefish *Euprymna scolopes* reference genome using HISAT and implemented in Trapnell et al. (2009) [26]. The number of uniquely mapped reads spanned from 88.08% (Ova I_2) to 95.90% (Ova III_1) and were mapped to the *E. scolopes* reference genome (Table 3). Furthermore, approximately > 88% clean reads were successfully mapped on to the *E. scolopes* reference genome. The extensive genome coverage achieved in our RNA-Seq analysis indicates the high quality of the collected samples.

**Table 3:** Sequencing data statistics of sixteen ovarian transcriptomes

Samples	Total reads	Clean reads	GC Content	> Q20 (%)	> Q30 (%)	Mapped reads	Mapping ratio (%)
Ova I_1	45,922,078	45,663,486	0.4094	0.9857	0.958	41,678,733	88.99
Ova I_2	43,970,414	43,681,416	0.4071	0.9849	0.9563	39,343,975	88.08
Ova I_3	43,337,798	43,022,676	0.4059	0.9846	0.9555	38,923,198	88.31
Ova I_4	41,589,862	41,302,638	0.4093	0.9852	0.9574	37,389,361	88.32
Ova II_1	48,047,478	47,748,664	0.3844	0.9854	0.9571	53,930,435	94.44
Ova II_2	52,488,460	52,055,714	0.3844	0.9829	0.9512	58,028,368	94.13
Ova II_3	47,016,870	46,766,228	0.385	0.9863	0.959	51,600,371	94.17
Ova II_4	45,766,492	45,497,180	0.3899	0.9861	0.9588	49,894,335	93.39
Ova III_1	42,944,820	42,623,226	0.4459	0.9863	0.9602	59,457,832	95.90
Ova III_2	45,370,062	45,149,468	0.3924	0.9882	0.9648	50,954,872	94.32
Ova III_3	45,187,798	44,999,066	0.4111	0.9894	0.9677	48,625,525	93.41
Ova III_4	48,053,906	47,766,224	0.4018	0.9775	0.9464	50,263,064	88.63
Ova IV_1	45,154,682	44,900,118	0.3931	0.9877	0.9637	52,531,740	94.97
Ova IV_2	46,250,816	46,006,972	0.4085	0.9831	0.9575	52,783,341	91.09
Ova IV_3	42,764,972	42,530,970	0.3964	0.9878	0.9639	50,315,187	94.86
Ova IV_4	41,091,122	40,890,772	0.4222	0.9875	0.9624	49,868,115	94.47

### Differential expression gene (DEGs) analysis of *S. pharaonis* ovarian samples at different stages

**Fig. 2:**
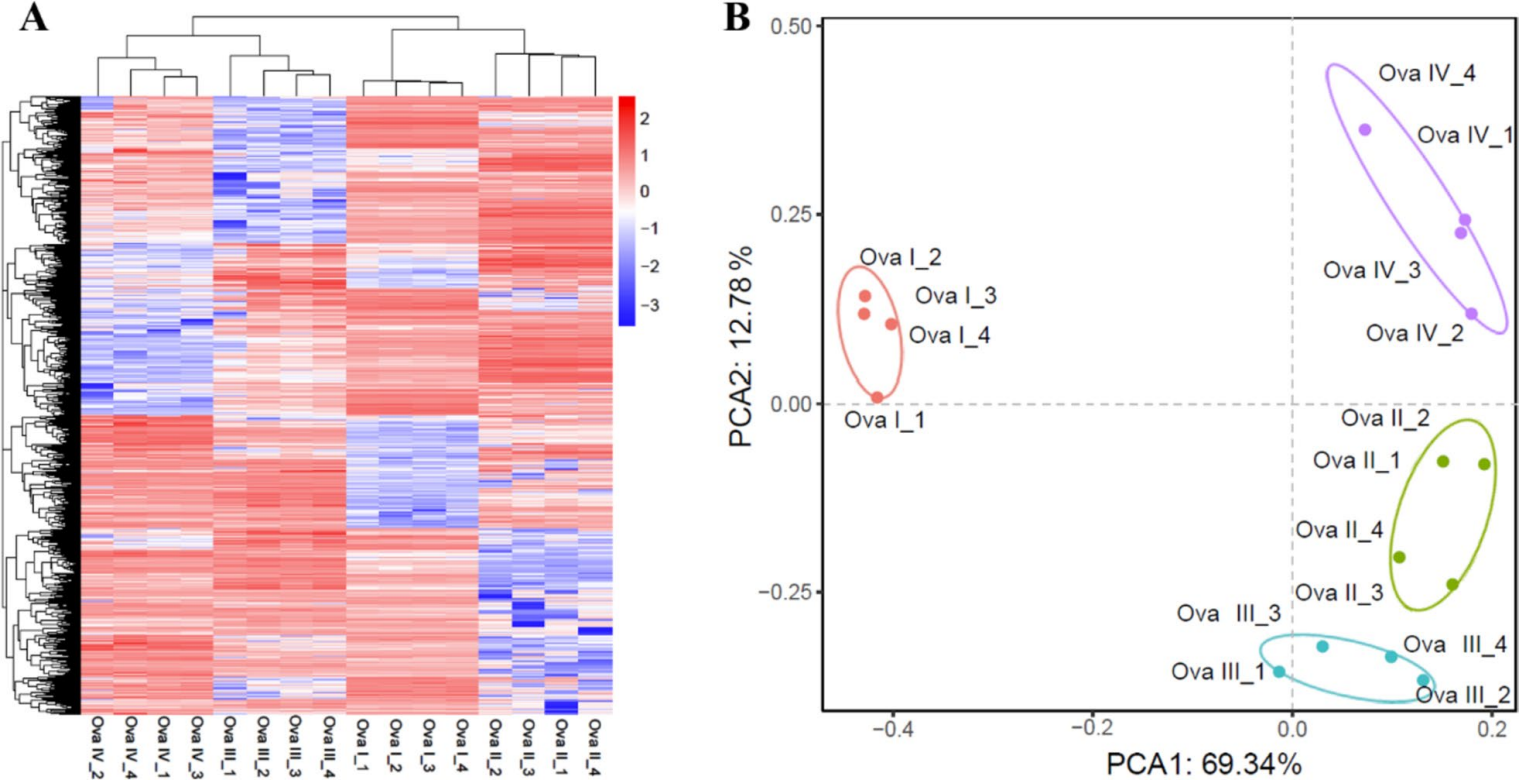
The gene expression pattern of DEGs of twelve ovary samples of *S. pharaonis*. **A **Hierarchical clustering for the DEGs among the sixteen ovarian samples. The blue color in the matrix represents downregulation of gene, the red color in the matrix represents upregulation of gene. **B **PCA of sixteen ovarian samples in different stages

A total of 136,829 differentially expressed genes (DEGs) were identified from the sixteen ovarian samples of *S. pharaonis* (Table S1). The results clearly distinguished the expression patterns of different stages of ovarian development, and the quadruplicate expression patterns were consistent at each stage (Fig. 2A). Principal component analysis (PCA) plots of the sixteen ovarian samples of *S. pharaonis*, with each circle representing a sample from a different ovarian developmental stage, highlighted the differences among the stages (Table S1). The profiles of the sixteen ovarian samples were categorized into four groups corresponding to each stage. In terms of ovarian development, *S. pharaonis* stages Ova II and Ova III showed the greatest similarity, whereas Ova I was the most distinct from the other stages (Fig. 2B).

### Overall functional annotation and analysis

To understand the functions of lineage-specific homologous genes in *S. pharaonis* from other species, we performed KOG analysis, classifying 8,689 annotated genes into 25 functional categories (Table S2).

**Fig. 3:**
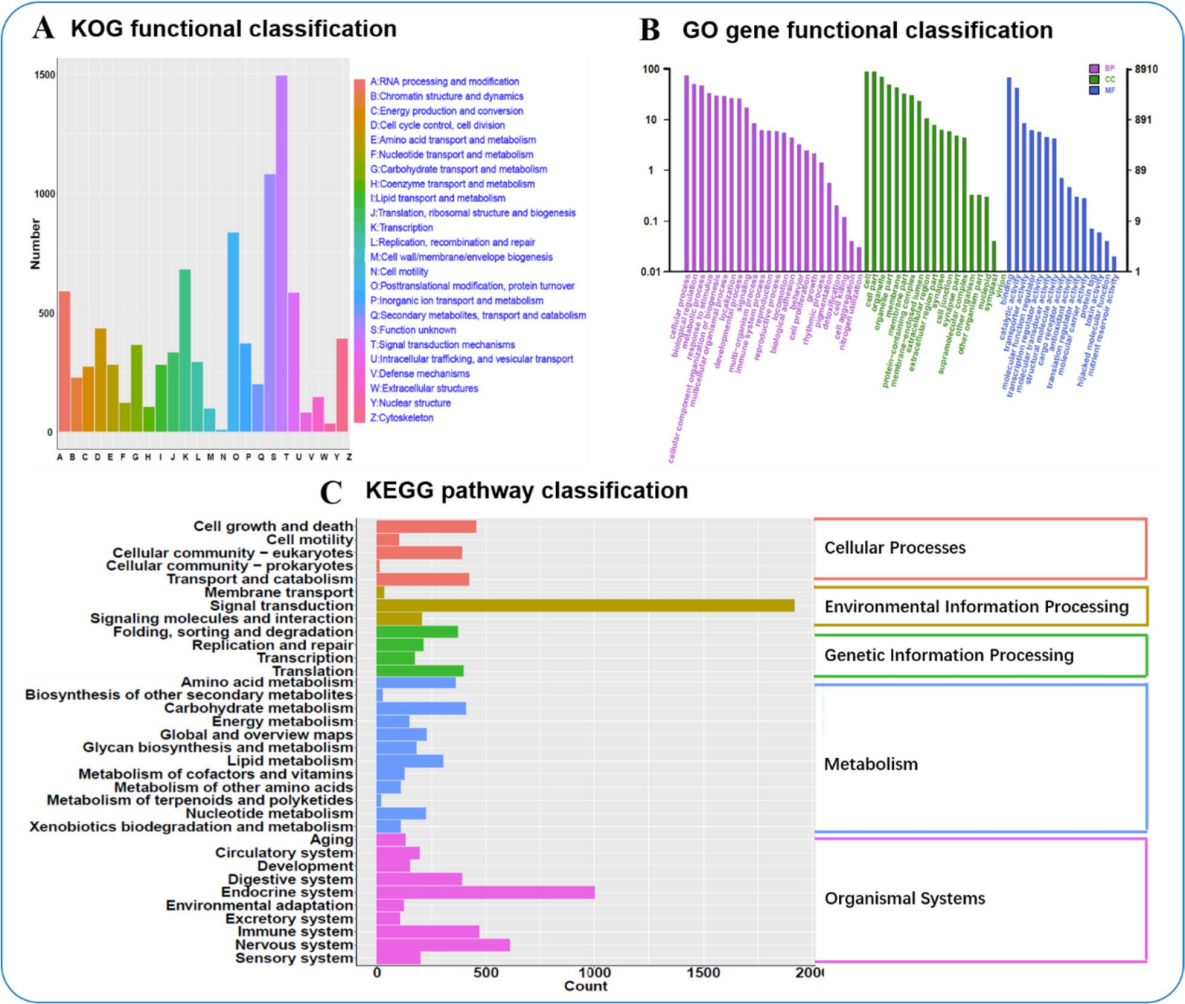
The overall functional annotation of the assembled genes. **A **the overall KOG functional classification of the genes. **B **the overall GO classification annotation of the genes. **C **the overall KEGG pathway classification of the genes

The results showed that the cluster of signal transduction mechanisms (1492 genes, 17.17%) was the most dominant in the ovary of *S. pharaonis*, followed by posttranslational modification, protein turnover, chaperones (883 genes, 9.58%), and Transcription (678 genes, 7.8%) (Fig. 3A). Furthermore, in the GO functional classification analysis, a total of 8910 genes were annotated to at least one GO term; all the GO annotated genes were classified into 59 GO terms belonging to the three main categories of molecular function, biological process, and cellular components (Table S3). The cellular component was the most frequently annotated category, and the cell (7805 genes, 87.6%), cell part (7773 genes, 87.26%), and organelle (6221 genes, 69.82%) were the most common subcategories (Fig. 3B). The biological process category was ranked second in the frequency of annotations, and the cellular process (6551 genes, 73.52%), biological regulation (4439 genes, 49.82%), and metabolic process (4163 genes, 46.72%) were the most common subcategories. From the molecular function category, the binding (6062 genes, 66.02%) and catalytic activity (3762 genes, 42.22%) were the most dominant subcategories. In the biological process category, the subcategories of reproduction and reproductive process manifested 530 (5.95%) and 526 (5.9%) genes, respectively. In addition, to understand the biological pathways of the genes, 13,211 genes annotated in the KEGG database were analyzed and were assigned to 311 pathways (Table S4). In the KEGG pathway analysis, categories such as cellular processes, environmental information processing, genetic information processing, metabolism, and organismal systems were identified. These pathways comprised 5, 3, 4, 11, and 10 subgroups, respectively. Among them, signal transduction, endocrine system, and nervous system were prominent (Fig. 3C).

### Comparative transcriptome analyses in six comparison groups

**Fig. 4:**
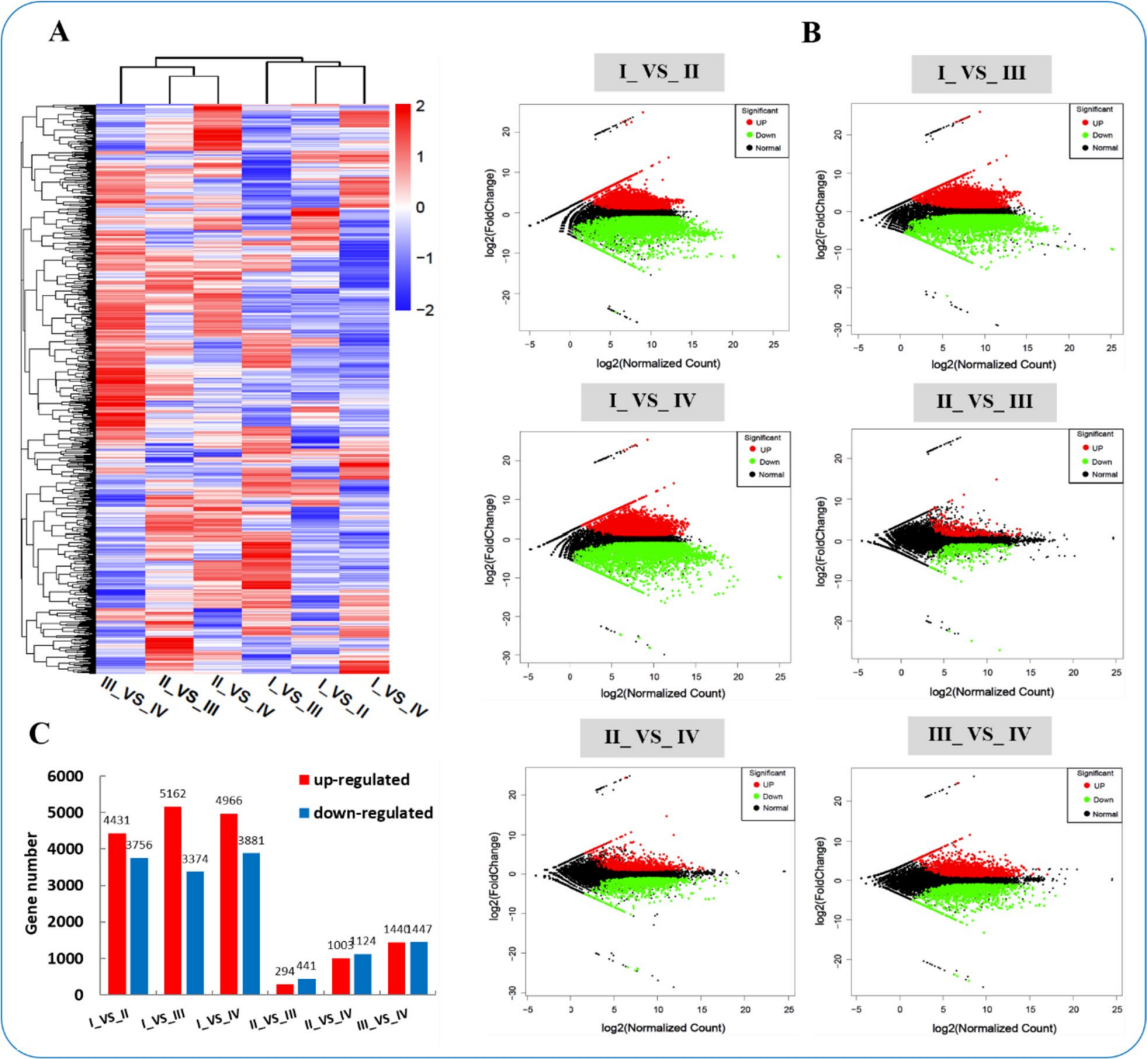
The analysis of expression differences in different comparison groups. **A **the six comparisons cluster heatmap of DEGs. The blue color in the matrix represents downregulation of genes, the red color in the matrix represents upregulation of genes. **B **The MA plot of the DEGs in six comparisons. **C **The bar charts of the DEGs numbers in six comparisons

The results of the DEG heat map analysis in the following comparison groups: Ova I_VS_Ova II, Ova I_VS_Ova III, Ova I_VS_Ova IV, Ova II _VS_Ova III, Ova II _VS_Ova IV, and Ova III_VS_Ova IV are shown in Fig. 4A and Table S5. These results indicated that the expression patterns of DEG genes in all six comparison groups were significantly different, especially in Ova I_VS_Ova IV. The MA plot listed the number of DEGs in each comparison group. The Ova I_VS_Ova II, Ova I_VS_Ova III, Ova I_VS_Ova IV, Ova II _VS_Ova III, Ova II _VS_Ova IV, and Ova III_VS_Ova IV comparisons had 4,431, 5,162, 4,966, 294, 1,003, and 1,440 significantly up-regulated genes, respectively. They also had 3,756, 3,374, 3,881, 441, 1,124, and 1,447 significantly down-regulated genes, respectively (Fig. 4B, C and Table S6).

### DEGs UpSetR plot and Venn diagram of six comparisons groups

**Fig. 5:**
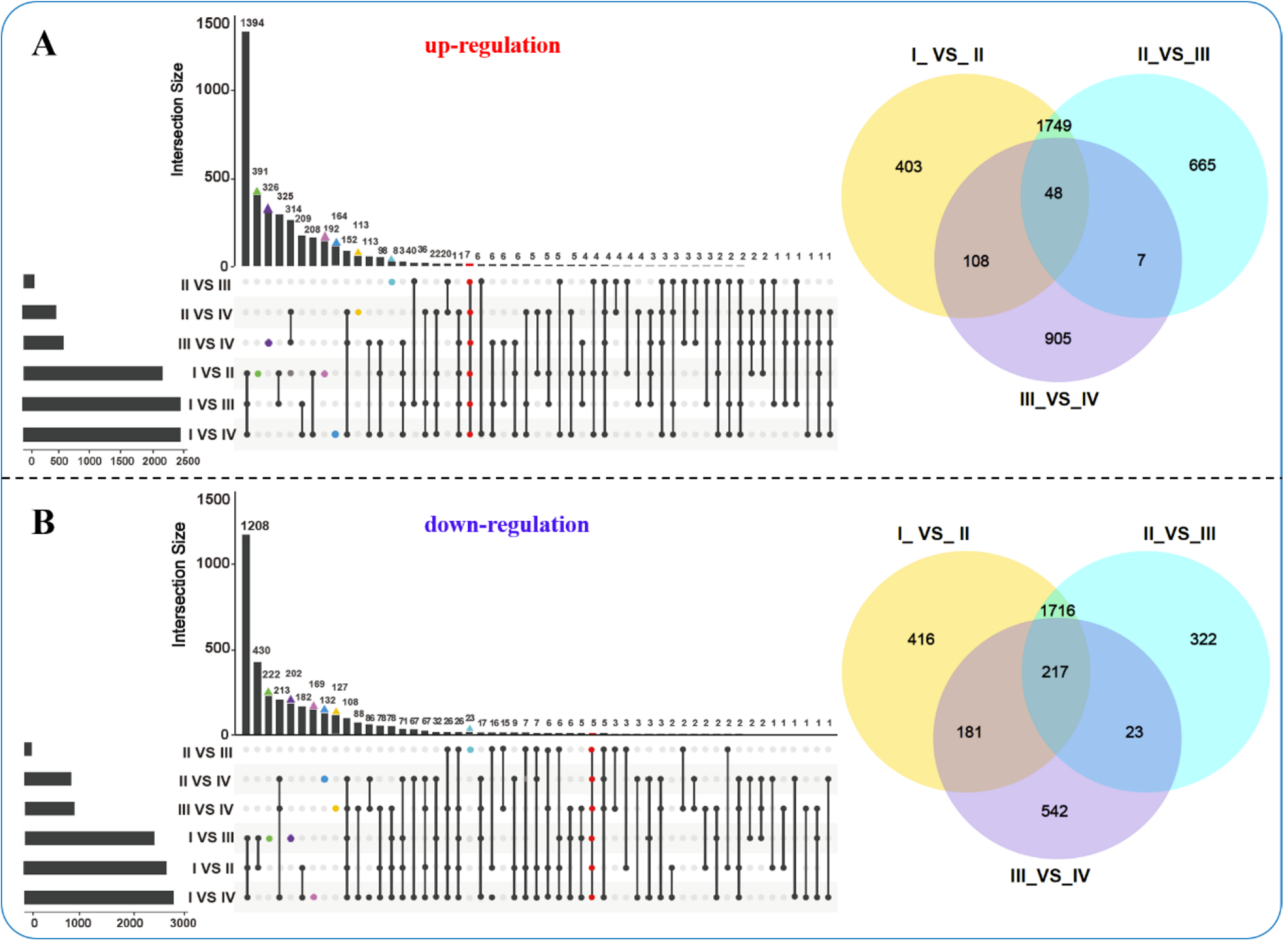
An UpSetR plot of six comparisons groups with intersection queries. Set metadata is plotted to the left of the set size bar. Statistics are based on the sum of quadruplicate in each stage. **A **The left panel is the UpSetR plot of the up-regulated genes, and the right panel is the Venn diagram of continuous ovarian development (Ova I_VS_Ova II, Ova II_VS_Ova III, and Ova III_VS_Ova IV). **B **The left panel is the UpSetR plot of the down-regulated genes, and the right panel is the Venn diagram of continuous ovarian development

As depicted in Fig. 5, an UpSetR plot was employed to interrogate the intersections of DEGs across various comparison groups. A total of 1,394 up-regulated DEGs were identified across the comparison groups Ova I versus Ova II, Ova I versus Ova III, and Ova I versus Ova IV, whereas 1,208 DEGs were concurrently down-regulated. Moreover, across the six comparison groups—Ova I versus Ova II, Ova I versus Ova III, Ova I versus Ova IV, Ova II versus Ova III, Ova II versus Ova IV, and Ova III versus Ova IV—seven DEGs were found to coexist (Fig. 5A). Additionally, five DEGs coexisted across the six comparison groups in the context of down-regulated genes (Fig. 5B). Further analysis was performed using a Venn diagram to observe the distribution of DEGs among the different stages of ovarian development. Among the up-regulated DEGs, there were 48 core genes (Fig. 5A, right panel), while 217 core genes were down-regulated in the continuous ovarian development comparison groups (Fig. 5B, right panel and Tables S7, S8).

### K-means clustering analysis of DEGs

**Fig. 6:**
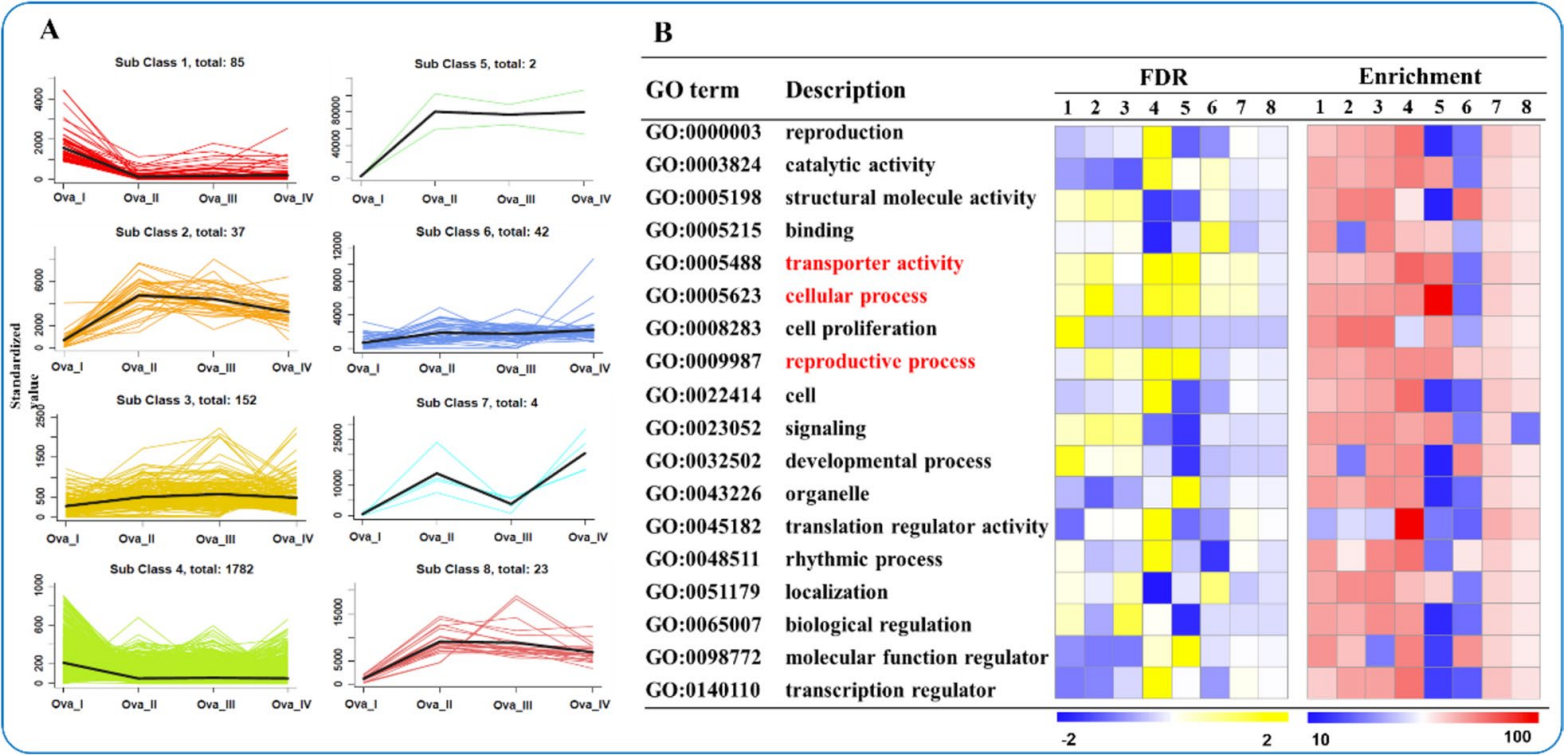
K-means clustering analysis of DEGs obtained from Ova_I to Ova_IV. **A **A total of eight clusters were identified using the K-means clustering analysis. **B **Functional annotation of the DEGs in the eight clusters

DEGs from four comparison groups were clustered via K-means analysis to elucidate the molecular mechanisms underpinning the various stages of ovarian development in *S. pharaonis*. Among the eight clusters, four (3, 5, 6, and 7) represented up-regulated genes and two (2 and 8) represented down-regulated genes across different stages of ovarian development (Fig. 6A and Table S9). GO enrichment analysis revealed that “transporter activity,” “cellular process,” and “reproductive process” were annotated in all clusters and distinctly altered in clusters 3, 4, and 5 (Fig. 6B). Other GO biological processes, such as reproduction in cluster 4, and binding and localization in cluster 6, were also identified based on the K-means clustering analysis.

### Functional annotation of differentially expressed genes

**Fig. 7:**
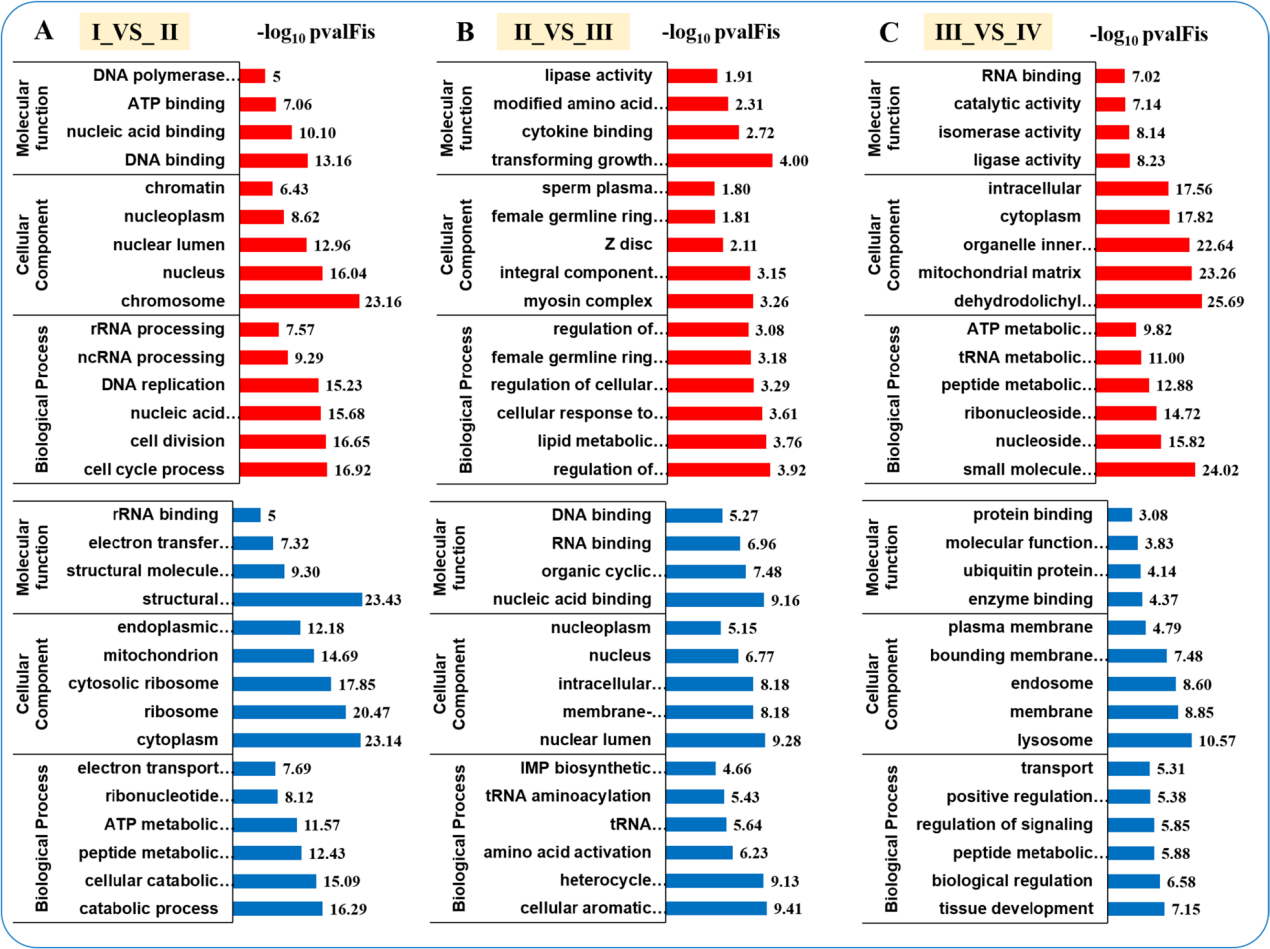
GO enrichment analysis of DEG comparison groups in continuous ovarian development. **A**, **B**, and **C** are GO enrichment of the Ova I_VS_Ova II, Ova II_VS_Ova III, and Ova III_VS_Ova IV, respectively. The red part of the plate is presented as GO enrichment of up-regulated genes. The blue part of the plate is presented as GO enrichment of down-regulated genes

DEGs were categorized and enriched in GO and KEGG functional classes to identify sets of genes with differential expression. The GO classification of DEGs revealed a distribution across three primary categories; notably, the number of DEGs in the Ova I versus Ova II (7,622 DEGs) and Ova II versus Ova III (7,902 DEGs) comparison groups were substantially greater than in the Ova III versus Ova IV (2,768 DEGs) group (Table S10). The up-regulated DEGs in the biological process category across all comparison groups were primarily associated with cell cycle processes, cell division, regulation of keratinocyte proliferation, small molecule metabolic processes, and tRNA metabolic processes. In the cellular component category, the up-regulated DEGs were predominantly related to terms such as chromosome, nucleus, dehydrodolichyl diphosphate synthase complex, and mitochondrial matrix. Regarding molecular function, the up-regulated DEGs were mainly located in terms such as DNA binding, ATP binding, transforming growth factor beta binding, and RNA binding in all comparison groups. Furthermore, the DEGs of most of the terms in Ova I_VS_Ova II, Ova II_VS_Ova III and Ova III_VS_Ova IV comparisons were dominated by up-regulation, while the DEGs in Ova IV_vs_Ova I and Ova IV_vs_Ova II comparisons were dominated by down-regulation, illustrating that the some GO terms increased from Ova I to Ova IV. Interestingly, in the category of biological processes of Ova I_VS_Ova II, there were 6.81% and 6.81% DEGs in the subcategories of reproductive and reproductive processes, respectively, and increased to 14.25% and 14.06% in Ova III_VS_Ova IV, respectively. This suggests that the ovaries develop rapidly in Ova III and Ova IV. Conversely, the down-regulated DEGs in the biological process category across all comparison groups were primarily associated with catabolic processes, cellular aromatic compound metabolic processes, and tissue development. In the cellular component category, the down-regulated DEGs were predominantly related to terms such as cytoplasm, nuclear lumen, and lysosome. Regarding molecular function, the down-regulated DEGs were mainly located in terms such as structural constituent of ribosome, nucleic acid binding, and enzyme binding in all comparison groups (Fig. 7 and Table S11).

**Fig. 8:**
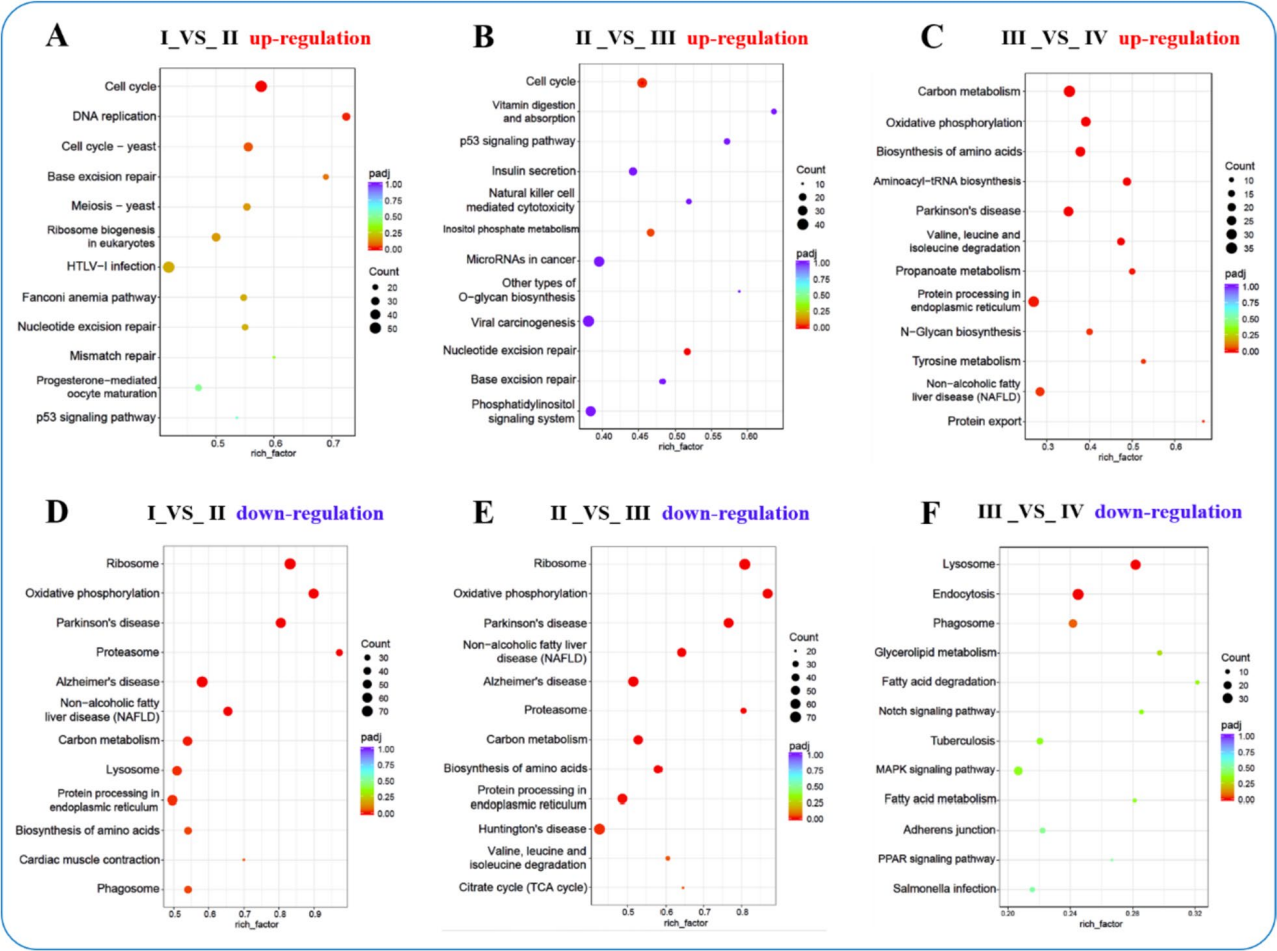
The analysis of KEGG pathway functional enrichment. The ordinate represents the top twelve enriched KEGG pathways, the rich factor is the proportion of the DEGs annotated in the KEGG pathway to the total annotated genes in the KEGG pathway. The redder color of the Q value, the more significant the enrichment of the KEGG pathway. The significant number of DEGs in the KEGG pathway is represented by the size of the circle. **A**, **B**, and **C** are the KEGG enrichment up-regulated DEGs of the Ova I_VS_Ova II, Ova II_VS_Ova III, and Ova III_VS_Ova IV, respectively. **D**, **E**, and **F** correspond to the KEGG enrichment down-regulated DEGs analysis

Additionally, KEGG classification revealed that DEGs were distributed across five major categories: cellular processes, environmental information processing, genetic information processing, metabolism, and organismal systems (Table S12). The environmental information processing category’s “Signal Transduction” subgroup was notably more prevalent in comparisons involving Ova I_VS_Ova II, Ova II_VS_Ova III, and Ova III_VS_Ova IV. The significant numbers of each subgroup in Ova I_VS_Ova II and Ova II_VS_Ova III were greater than in the Ova III_VS_Ova IV comparison, consistent with the analysis of the corresponding GO categories. Furthermore, the KEGG enrichment analysis of up-regulated DEGs revealed three significantly enriched subgroups: cell cycle, DNA replication, and p53 signaling pathway. These subgroups were observed in the Ova I_VS_Ova II and Ova II_VS_Ova III comparison groups, indicating their involvement in the transition from stage II to stage IV. In contrast, the significantly enriched subgroups in the Ova III_VS_Ova IV comparison were carbon metabolism, biosynthesis of amino acids, and aminoacyl-tRNA biosynthesis, further supporting their role in this transition (Fig. 8 and Table S13). Regarding the down-regulated DEGs, the most enriched subgroup in the Ova I_VS_Ova II and Ova II_VS_Ova III comparison groups was ribosome. In contrast, the Ova III_VS_Ova IV comparison showed enrichment in the lysosome and endocytosis subgroups (Fig. 8 and Table S13).

### Screening candidate genes for regulating ovarian development

We also conducted a detailed analysis of the functional and metabolic pathways of DEGs, identifying differential expression patterns during ovarian development. Among the KEGG pathways in Ova I_VS_Ova II, Ova II_VS_Ova III, and Ova III_VS_Ova IV comparison groups, we focused on the reproduction and endocrinology biosynthetic pathways associated with ovarian development. Moreover, four KEGG pathways—steroid hormone biosynthesis, GnRH signaling, ovarian steroidogenesis, and neuroactive ligand-receptor interaction—were found to be enriched across the four developmental stages. In total, eight candidate genes were annotated to nine GO terms, including estrogen biosynthetic process, estradiol 17-beta-dehydrogenase activity, estrogen receptor binding, progesterone receptor signaling pathway, growth hormone synthesis, vitellogenesis, neuropeptide F receptor activity, and neuropeptide Y receptor activity (Table 1).

### Verification of the expression patterns of ovarian developmental regulatory genes by RT‑qPCR

**Fig. 9:**
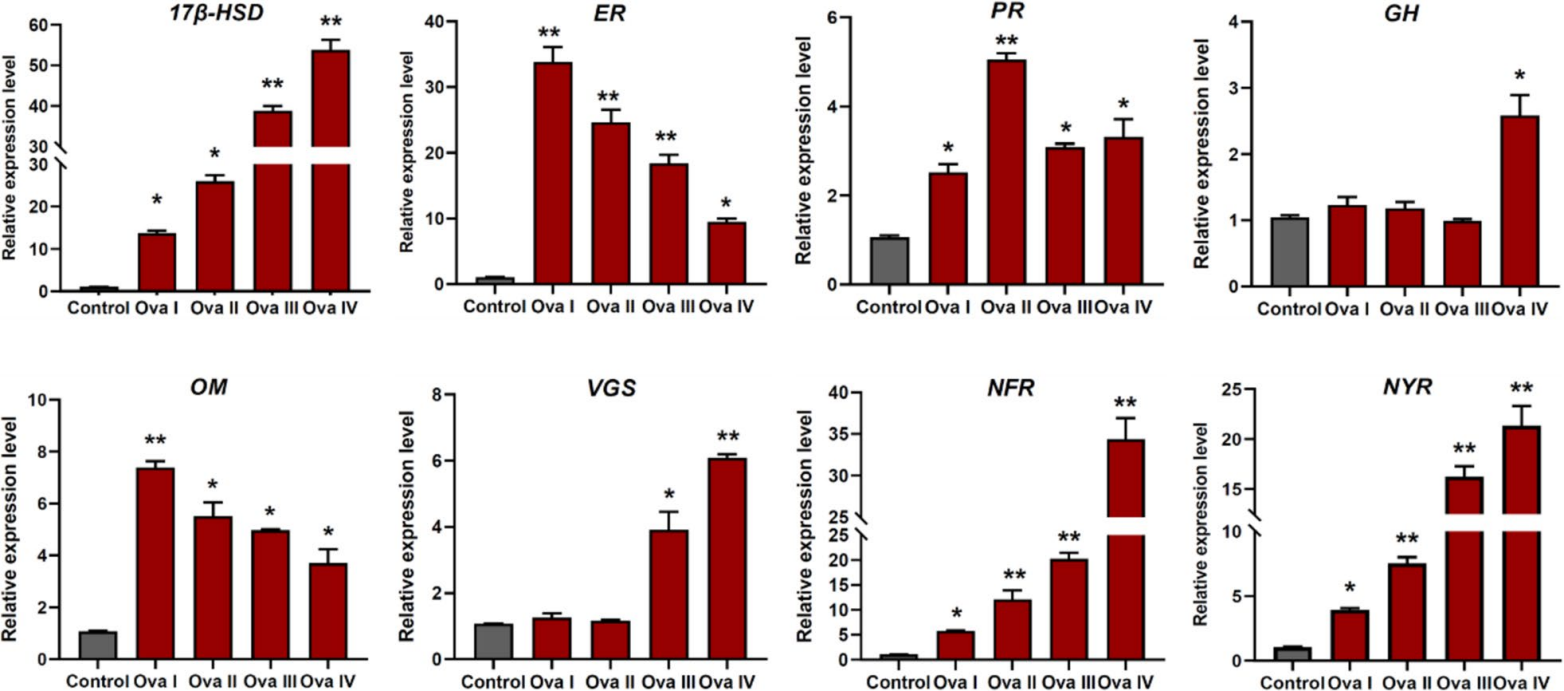
The relative expression level of *17β-HSD*, *ER*, *PR*, *GH*, *OM*, *VGS*, *NFR* and *NYR* in ovary of *S. pharaonis* at four ovarian development stages. β-actin was used as the control. The data are presented as the means ± S.D relative expression level. * Significant difference (*P* < 0.05), ** Extremely significant difference (*P* < 0.01)

In summary, eight candidate genes related to reproduction and endocrinology—namely Estradiol 17-beta-dehydrogenase (*17β-HSD*), Estrogen receptor (*ER*), Progesterone receptor (*PR*), Growth hormone (*GH*), Oocyte meiosis (*OM*), Vitellogenesis (*VGS*), Neuropeptide F receptor (*NFR*) and Neuropeptide Y receptor (*NYR*)—were isolated from the DEGs and subjected to RT-qPCR to validate their expression patterns in *S. pharaonis*. The expression levels of the eight genes were significantly different at four developmental stages (Fig. 9). The expression of *17β-HSD* increased sharply with ovarian development and maturation, reaching the highest level in Ova IV, approximately 53-fold higher than the control group (*P* < 0.01). In contrast, *ER* showed a negative correlation with ovarian maturation, with significantly decreased expression levels as ovarian development progressed. The highest expression level of *ER* was observed in Ova I, approximately 33-fold higher than the control group (*P* < 0.01). *OM* exhibited a similar expression profile to *ER*. *PR* displayed dominant expression in Ova II (*P* < 0.01), while *GH* was only highly expressed in Ova IV, without significant differences between the control group and the other three developmental stages. The expression patterns of *VGS*, *NFR*, and *NYR* were consistent, with gradual increases in expression as ovarian development progressed. The highest expression levels of these genes were observed in Ova IV (*P* < 0.01).

## Discussion

Historically, *S. pharaonis* was among the four predominant traditional fisheries in China. Meanwhile, *S. pharaonis* possesses great aquaculture potential, attributable to its high nutritional value, rapid growth, large body weight (wild individuals exceeding 5 kg), short life cycle, and fast generational turnover (1-year lifespan). However, fundamental biological research on *S. pharaonis* and cephalopods, in general, remains relatively weak, particularly regarding the reproductive system of female cephalopods. In recent years, issues such as low egg production, poor egg quality, weak physical condition of newly hatched juveniles, and low survival rates, have emerged during the development of full captive breeding of this species. Clarifying these issues may serve as a prerequisite for overcoming key technological bottlenecks in cephalopod aquaculture. The ovarian development of female *S. pharaonis* is a crucial physiological process that represents reproductive performance. Although we have elucidated the characteristics of oogenesis and ovarian development in this organism, the overall reproductive mode of *S. pharaonis* remains largely unknown, especially the molecular mechanisms underlying ovarian development. This lack of knowledge significantly hampers its production and application. To gain further insights into the molecular mechanisms underlying ovarian development, we employed Illumina HiSeq X Ten sequencing to obtain transcriptomic data from four developmental stages of *S. pharaonis* ovaries. We conducted comparative analyses between these developmental stages to elucidate the patterns of ovarian development.

In the present study, we conducted transcriptomic analyses on samples ovarian samples across four developmental stages. The total clean read counts of the sixteen samples ranged from 40,890,772 (Ova IV_4) to 52,055,714 (Ova II_2). The total number of clean bases ranged from 5,958,274,972 bp (Ova IV_4) to 97,653,060,592 bp (Ova II_2). These values were similar to the clean reads obtained from immature and mature gonads of *Sepiella japonica*, which were 53,116,650 and 53,446,640 bp, respectively [10] (Lü et al., 2016). The average GC content in our study ranged from 38.44 to 44.59%, differing from the 37.45% reported by Zhang et al. (2023) [8]. We had a larger number of samples and a higher Q30% (> 95%) compared to the other study. This suggests that the GC ratio in our results is more reliable. We conducted correlation analysis on the 16 ovarian samples based on RNA-seq data. The Pearson correlation coefficients showed a strong correlation (> 0.7) among the four samples from the same stage, indicating a strong correlation within each group. The Pearson coefficients also demonstrated that stage I was distinct from the other stages, while stage II was similar to stage III. Furthermore, PCA analysis confirmed the distinguishability of each stage, supported by a significant Anosim *R*-value of 0.6132 (*P* < 0.001). These results indicate that the grouping of the four developmental stages in this study was reliable. The DEG facilitated the identification of genes with differential expression across diverse samples, providing insights into the relationship between genotype and phenotype. Lü et al. (2016) [12] used Illumina HiSeq 2000 technology to identify a total of 793 immature ovary-AEGs and 38 mature ovary-AEGs in the transcriptome of *S. japonica*. However, the number of identified DEGs was relatively low. In our study, we employed next-generation sequencing technology and identified a larger number of DEGs between early vitellogenic ovaries (stage II) and pre-vitellogenic ovaries (stage I), thereby providing additional information on cuttlefish ovarian development. Additionally, we observed that the difference between mature ovaries (stage IV) and pre-vitellogenic ovaries (stage I) was greater compared to the other groups, while the difference between mid-vitellogenic ovaries (stage III) and early vitellogenic ovaries (stage II) was smaller compared to the other groups.

The GO functional classification allows for the analysis of functional categories assigned to individual genes or differentially expressed genes (DEGs) in transcriptomic analysis. For S. esculenta, the most assigned biological processes were related to reproduction and reproductive processes [8]. In this study, we performed a GO functional classification analysis on the comparison groups during ovarian development (Ova I_VS_Ova II to Ova III_VS_Ova IV).

We observed that the upregulated DEGs in these groups were mainly involved in the cell cycle process, cell division, regulation of keratinocyte proliferation, small molecule metabolism process, and reproduction. Additionally, we identified genes related to steroid biosynthesis (GO:0006694), progesterone receptor (GO:0050847), estradiol 17-β dehydrogenase (GO:0004303), and neuropeptide receptor (GO:0008188) that were differentially expressed in the comparison groups. Previous studies have associated these genes with reproductive processes, cell signal transduction [27–29], and neuropeptide receptor (GO:0008188) gene had been characterized to modulate cell growth, apoptosis, proliferation and migration [30]. Studies have confirmed their direct or indirect involvement in the regulation of reproductive processes in bivalves, while also playing a crucial role in yolk formation, oogenesis, and secretion of eggs [31, 32]. These findings are also consistent with the transcriptome results of Lü et al. (2016) [33] in female *S. maindroni*. In addition, we also observed significant expression of certain genes during the early stages of yolk formation in the ovary. These genes include the initiation of primordial ovarian follicle growth gene (GO:0001544), sex differentiation gene (GO:0007548), and transforming growth factor beta receptor gene (GO:0005025). They are potentially involved in the regulation of early ovarian development. During the oocyte formation period, several genes exhibited significant expression compared to the early yolk formation stage. These genes included the insulin-like growth factor receptor gene (GO:0043567), estrogen receptor gene (GO:0030331), ovulation from ovarian follicle gene (GO:0001542), and ovarian follicle development gene (GO:0001541). These genes are likely to directly participate in the regulation of oocyte development and oogenesis. Overall, our findings indicate that different stages of *S. pharaonis* ovarian development involve different pathways.

KEGG analyses were utilized to identify molecular metabolic pathways involved in various processes [34]. In this study, KEGG-enriched upregulated DEGs analysis exhibited significant enrichment of subsets during ovarian development (Ova I_VS_Ova II to Ova III_VS_Ova IV), including the cell cycle, DNA replication, p53 signaling pathway, carbon metabolism, amino acid biosynthesis, and aminoacyl-tRNA biosynthesis. Furthermore, the cell cycle pathway, steroid hormone biosynthesis pathway, GnRH signaling pathway, insulin signaling pathway, and TGF-beta signaling pathway were identified, which are associated with gonadal development, maturation, signal transduction, endocrine system, and reproduction, as demonstrated in previous studies [8, 35–37]. The cell cycle, insulin, and GnRH signaling pathways are involved in gonadal development, ovarian steroidogenesis, oocyte maturation, and steroid hormone biosynthesis [8, 37–39], indicating their potential regulatory roles in follicle development in *S. pharaonis*. The transforming growth factor-beta (TGF-β) signaling pathway plays a crucial role in regulating various cellular processes, including cell growth, differentiation, apoptosis, extracellular matrix production, activation, and growth of embryonic follicles [40]. Additionally, studies have shown that neuropeptides such as FMRFamide, neuropeptide F receptor, and neuropeptide Y receptor play various physiological roles in mollusks, including activating intracellular signaling pathways, protecting cells from apoptosis, and participating in reproductive regulation [41–44]. These neuropeptides are predominantly found in the central nervous system or neural tissues [45]. The K-means clustering results showed that the GO-enriched term “cellular process” was annotated in cluster 5, exhibiting significant upregulation during ovarian development. Notably, cluster 5 contains only two genes (new_gene1040 and new_gene3254). Further analysis of these genes revealed that new_gene3254 is a precursor to egg-yolk proteins (vtg1). Vitellogenins (vtgs) are precursors to yolk proteins in almost all female oviparous animals, including fish, reptiles, amphibians, and birds. It is widely accepted that vtgs play a vital role in transporting nutrients to the ovary [46]. Consequently, vitellogenesis is highly correlated with nutrient metabolism, particularly lipid processes. For instance, vtgs are associated with ovarian fatty acid synthesis. This was observed in yellowtail rockfish (*Sebastes flavidus*), which displayed significantly increased concentrations of polar lipids, triacylglycerols (TG), and cholesterol in the ovary during late vitellogenesis [47]. In zebrafish, the transcription levels of vtg1 mRNA are approximately 100 and 1000 times higher than those of vtg2 and vtg3 mRNAs, respectively, making vtg1 the most crucial vtg in this species [48]. Moreover, the absence of vtg1 in female zebrafish broodstock hinders the transportation of docosahexaenoic acid-enriched phosphatidylcholine (DHA-PC) from the liver to the ovary, subsequently reducing the DHA-PC content in the ovaries during the larval stage and in the offspring [49]. This suppression of vtg1 may compromise egg vitality, thereby adversely affecting embryonic development. Vtg is known to be the primary carrier for both DHA and EPA, with PC reported to be the main carrier of EPA in the gonads of striped bass [50]. It has been established that PC is the principal transporter of DHA from the liver to the ovary in female zebrafish, with vtg1 being responsible for this DHA-PC transport [49]. In summary, vtgs and DHA-PC play a critical role in the regulation of fatty acid transport during the reproductive process, significantly influencing oocyte maturation and vitellogenesis. These processes, in turn, impact embryonic development and the quality of offspring juveniles. These findings offer new perspectives in understanding the challenges of low egg production, and poor egg quality in captive-bred *S. pharaonis*.

Notably, the expression levels of growth hormone and vitellogenesis were significantly higher in Ova IV compared to the early stages of yolk formation (Ova I and Ova II), as shown in Fig. 9. This elevation corresponds with the GnRH signaling pathway and ovarian steroidogenesis pathway, suggesting that these pathways may regulate oogenesis and oocyte maturation in *S. pharaonis* by sensing the levels of growth hormone and vitellogenesis. Similar findings have been reported in transcriptome analyses of different developmental stages of *Procambarus clarkii* ovaries [51]. Future studies will involve independent functional analyses of these differentially expressed genes (DEGs) to identify their roles in ovarian maturation and to elucidate the synergistic interactions among these reproductive regulatory genes. Additionally, forthcoming research will focus on comparing different transcripts (between wild and cultured specimens), selecting specific genes, and establishing cascading expression patterns of these genes (spanning from ovarian to embryonic development, and subsequently to juvenile development). These investigations could provide insights into issues such as low egg production, poor egg quality, weak juvenile fitness, and low survival rates under captive conditions.

## Conclusion

In summary, the first transcriptome profile of ovarian tissue in *S. pharaonis* was generated using the Illumina HiSeq X Ten sequencing technology, capturing four distinct stages of ovarian maturation: undeveloped stage (Ova I), developing stage (Ova II), nearly-ripe stage (Ova III), and ripe stage (Ova IV). The results of the comparative analyses revealed a V-shaped pattern in the number of differentially expressed genes (DEGs) identified during ovarian development. Specifically, the comparisons Ova I_VS_Ova II, Ova II_VS_Ova III, and Ova III_VS_Ova IV yielded 8187, 735, and 2887 DEGs, respectively. GO analysis highlighted the involvement of ovarian follicle growth, sex differentiation, and transforming growth factor beta receptor in the early stages of ovarian development, while small molecule metabolic process, peptide metabolic process, and catalytic activity were prominent in the mature stage. KEGG analysis unveiled that the early ovarian development of *S. pharaonis* was primarily associated with the cell cycle, DNA replication, and carbon metabolism pathways, whereas mid-late ovarian development involved signal transduction, endocrine system, and reproductive pathways. These findings provide valuable insights into the molecular mechanisms underlying ovarian development in *S. pharaonis* and have the potential to contribute to enhancing the reproductive performance of this species.

### Supplementary Information

**Additional file 1.**

**Additional file 2.**

**Additional file 3.**

**Additional file 4.**

**Additional file 5.**

**Additional file 6.**

**Additional file 7.**

**Additional file 8.**

**Additional file 9.**

**Additional file 10.**

**Additional file 11.**

**Additional file 12.**

**Additional file 13.**

## Data Availability

All data generated or analyzed during this study are available in this article and its supplementary information files. The transcriptomic raw data in the current study have been deposited to the Mendeley database with the accession DOI of 10.17632/87hwwbj96j.2, (https://data.mendeley.com/drafts/87hwwbj96j).
